# A meta-analysis of the efficacy and safety of the traditional Chinese medicine formula Kaixinsan decoction for depression

**DOI:** 10.1097/MD.0000000000036719

**Published:** 2024-01-05

**Authors:** Jia-liang Li, Lin Lin, Min-min Wu, Jing-yu Zhang, Yi-xin Zhang, Meng-ru Cao, Long Wang

**Affiliations:** a Heilongjiang University of Chinese Medicine, Harbin, China; b Department of Gynecology and Obstetrics, First Hospital of Harbin Medical University, Harbin, China; c Fujian University of Chinese Medicine, Fuzhou, China; d The Second Department of Mammary Gland, Harbin Medical University Cancer Hospital, Harbin, China; e The Third Department of Acupuncture, First Affiliated Hospital of Heilongjiang University of Chinese Medicine, Harbin, China.

**Keywords:** Chinese traditional medicine, Kaixinsan, meta-analysis, side-effects reduction, treatment efficacy, treatments for depression

## Abstract

**Background:**

Kaixinsan (KXS) decoction is a traditional Chinese herbal formulation commonly used to treat depression. This study aimed to evaluate the efficacy and safety of KXS, which is widely used, alone and in combination with other therapies, for the treatment of depression. The main objective was to assess the efficacy and safety of KXS in the treatment of depression as a single agent or in combination with other methods.

**Methods:**

Randomized controlled trials of KXS in the treatment of depression were systematically searched from several Chinese and English databases with no language restriction. Patients in these studies met the relevant diagnostic criteria for depression. Data on HAMD, SDS, practical situations, and occurrence of side effects in the studies were extracted. Finally, the methodological quality and risk of bias of the included studies were assessed using the Jadad scale and Cochrane bias evaluation tool.

**Results:**

Twelve studies with 1034 patients were included after screening. The Jadad scale and Cochrane bias evaluation tool indicated that the quality of the studies ranged from fair to good, with 41.7% categorized as good and 58.3% as poor. Egger test and funnel plots showed that the publication bias remain low.

**Conclusion:**

The results showed that the frequency of side effects in the control group was higher than that in the treatment group, and there was a statistically significant difference. KXS was comparable or superior to antidepressants in treating depression and has fewer side effects. The data analysis showed that effectiveness and other indicators differed significantly by geographic area and dosage form, which has implications for future clinical work.

## 1. Introduction

Depression is a common mental disorder. The core manifestations of low mood, loss of interest, and lack of pleasure, often accompanied by specific physical symptoms, significantly reduce a patient quality of life and ability to work, and in severe cases, may even produce suicidal intent.^[1]^ According to recent epidemiological surveys, in 2020, there are 3152.9 cases per 100,000 people who suffered from severe depression; that is, 246 million people will suffer from the disease worldwide.^[2]^ In addition, the prevalence rate among the adolescent population has increased from 24% to 37% in the last decade (2010–2020), and suicide rates are increasing every year.^[3,4]^ Rapid, effective, and safe treatment methods are required to relieve patients’ pain and restore their ability to live and work as early as possible.

Research in the 20th century has shown a direct relationship between serotonin and the onset of depression through the regulation of appetite, sleep, memory, learning, body temperature, and social behavior.^[5]^ Kraus et al also found that serotonin enhances nerve cell plasticity.^[6]^ To this day, many classic antidepressants are developed from this aspect. Cortisol is an important chemical that regulates mood, and the excessive release of cortisol due to stress is closely related to the development of depression.^[7–9]^ Chronic stress in patients with depression leads to lower serotonin levels and higher cortisol levels, which interact with each other to cause a vicious cycle of inflammation in the patient body, thereby exacerbating the condition.^[10,11]^

Treatments for depression usually include medication, psychotherapy, and physical therapy.^[12–14]^ Antidepressant drugs, the most widely used treatment, reap the benefits of efficacy accompanied by more side effects, such as dizziness, nausea, upper gastrointestinal bleeding, sexual dysfunction, and weight change,^[15–17]^ and patients are also more likely to experience relapse or rebound of symptoms after stopping the drug.^[18]^ In addition to single-use medication, treatment can be individually combined with psychotherapy, and antidepressant medication combined with psychotherapy tends to achieve better results.^[19,20]^ However, psychotherapy is more costly; it requires sufficient cooperation from the patient and demands higher compliance (the duration of treatment and the period of treatment are relatively long). There is still a lack of specialized psychotherapists in many regions; therefore, there is a need to find a wider variety of alternative therapies.

Traditional Chinese Medicine has gained popularity as a widely used complementary and alternative therapy for the treatment of depression,^[21]^ and Xiao Yao San, Gan Mai Da Zao Tang, Chai Hu Shu Gan San, and Kaixin San are commonly used formulas for treating depression. Systematic evaluations of the efficacy and safety of Xiaoxia San,^[22]^ Gan Mai Da Zao Tang,^[23]^ and Chai Hu Shu Gan San^[22]^ exist, but evaluations of Kaixinsan (KXS) are still lacking.

KXS originated in the Northern Zhou Dynasty, and in the Tang Dynasty, Sun Simiao recorded it in his work “Bijie Qianjin Yaofang” and specified the main ingredients as Renshen, Yuanzhi, Fuling, and Shichangpu in the ratio of 2:2:10:5. Over time, many KXS-type formulas (e.g., Dingzhi Wan and Dingzhi Xiaowan) have appeared with changes in the measurement ratios.^[24]^ However, the primary medicines were all the same, with additions to the original formula. According to previous reports, KXS, a combination of these herbs, can treat forgetfulness, depression, sadness, and insomnia. According to Chinese medicine, the cause of depression is the loss of nourishment of the heart and spirit and the deficiency of yang qi. The patient body is weak and inadequate emotions accumulate, leading to disorders in the 5 viscera and 6 bowels, first affecting the liver. The liver loss of detachment, inability to regulate the whole body qi, the further accumulation of destructive emotions, depression, loss of interest, and qi imbalance lead to the heart and kidney qi being unable to communicate; the spleen function weakens, resulting in a patient with fatigue and drowsiness, but can also lead to the spleen being unable to adjust the metabolism of the water and fluid. Water accumulates more in phlegm thereby obscuring the clear orifices, and then generates the symptoms of forgetfulness. In KXS, Renshen and Yuanzhi can nourish the heart and tranquilize the mind, replenish qi, and strengthen the spleen, while Shichangpu strengthens the spleen, reducing dampness, tranquilizing the heart, and benefitting the intellect. The efficacy of KXS is intended to nourish the heart and open the orifices, strengthen the spleen, tranquilize the mind, and transport the heart and kidneys; it has often been used to treat patients with depression in ancient and modern times.

In recent years, clinical trials of KXS for treating depression^[25–27]^ have begun to appear, and the results have shown that KXS has the same therapeutic effect as antidepressant drugs but is superior in terms of clinical side effects. Given that KXS has been widely used in the treatment of depression, it is necessary to conduct a systematic evaluation and meta-analysis into its potential role in the treatment of depression. This study aimed to assess the efficacy and safety of KXS for the treatment of depression.

## 2. Methods

### 2.1. Ethical notice

This study did not require ethical approval because no original data were collected in this review.

### 2.2. Search strategy

Four Chinese (CNKI, VIP, WANFANG, and Sinomed) and 4 English databases (PubMed, Embase, Web of Science, and the Cochrane Library) were searched from the time of their establishment to January 2023. Relevant references were collected from publications and studies not included in the electronic databases were also searched. We used the following combination of search strategies in the English databases: (“random*” OR allocation OR “random allocation” OR placebo OR single blind OR single blind method OR double-blind OR double blind method OR “randomized controlled trial*” OR “randomized controlled trial*” OR “RCT” OR “clinical trial*”) AND (kaixinsan OR kaixin* OR kai-xin-san OR kai-xin* OR KXS OR dingzhixiaowan OR dingzhi*) AND (Depressive Symptoms OR depression OR Depressive Symptom OR Symptom, Depressive OR Symptoms. Depressive OR Emotional Depression OR Depression, Emotional). The same Chinese terms were used to search the Chinese database. This systematic review and meta-analysis followed the PRISMA Reporting Items for evaluation.^[28]^

### 2.3. Eligibility criteria

The central inclusion in this study was a randomized controlled trial, and the patients participating in the trial met one of the following diagnostic criteria for depression: Diagnostic and Statistical Manual of Mental Disorders, 4th Edition (DSM-IV); Chinese Classification and Diagnostic Criteria of Mental Disorders, 3rd Edition Chinese classification of mental disorders (CCMD-3), International Classification of Diseases 10th edition (ICD-10), or other relevant standards; the patient treatment regimen consisted of the following: KXS and antidepressant medication were used separately, KXS and antidepressant medication were used alone, KXS and antidepressants versus antidepressants alone, or KXS and conventional nursing care or psychotherapy versus antidepressants versus conventional nursing care or psychotherapy, but excluding comparative experiments of KXS versus KXS in combination with antidepressants because the primary subject of the current study was KXS. In addition, at least one of the following outcome measures was available in the study: HAMD (Hamilton Rating Scale for Depression), SDS (Self-Rating Depression Scale), and treatment effectiveness; the secondary outcome was the occurrence of adverse events. For each included study, we extracted information on the study design (type of study, region, and diagnostic criteria), patient characteristics (including age and sex), design of the controlled experimental protocol (duration of treatment, dosage form, composition of KXS soup, and control interventions), and various pre- and post-treatment observables.

### 2.4. Study selection

The articles and extracted data were extracted separately and independently. Table 1 summarizes the details of the retrieved articles. Data characterizing the studies (first author, year of publication, country, experimental design, sex ratio, sample size, and treatment duration) were also collected. During data extraction, any conflicts or ambiguities in the reported methods or results were discussed with a third-party reviewer and resolved by consensus.

**Table 1:** Randomized controlled trials of KXS decoction for depression.

Participant characteristics									Intervention protocol				
Study ID	Country	Study design	Diagnostic system	Outcome measure	Adverse events (e.g./CG)	Jadad score	N (e.g./CG) % female	Mean age (SD)	Design	Treatment intervention	Control intervention	Period
Bao,2011	China	RCT	CCMD-3	78 (40/38) 67.95%	e.g.:41.24 (9.08) CG:42.13 (10.32)	2 parallel arms (KXS; AD)	KXS	Fluoxetine 20 mg/d	6 wk	Effective rate;	Adverse drug reactions (0/2)	3
Chen,2014	China	RCT	CCMD-3	80 (42/38) 46.25%	e.g.:65.43 (11.34) CG:64.42 (11.86)	2 parallel arms (KXS plus AD; AD)	KXS	Fluoxetine 20 mg/d	8 wk	HAMD; Effective rate;	Dry mouth (2/5) Abdominal distention (1/0) Constipation (0/3) Dizziness (0/2)	1
Hu,2021	China	RCT	DSM-IV	129 (63/66) 64.34%	e.g.:47.14 (14.29) CG:46.03 (11.92)	2 parallel arms (KXS plus AD placebo; AD plus KXS placebo)	KXS plus Fluoxetine placebo	Fluoxetine 20 mg/d plus KXS placebo 3.2 g/d	8 wk	HAMD; SDS;	Adverse drug reactions (3/10) Gastrointestinal reaction (2/5) Granulopenia (0/1) Xerostomia (2/2) Hyperlipemia (1/1) Dizziness (1/1) Fatigue (0/1) Headache (0/1)	6
Huang,2019	China	RCT	ICD-10	100 (69/31) 77.00%	e.g.:45.48 (12.30) CG:45.77 (7.81)	2 parallel arms (KXS; AD)	KXS	Fluoxetine 20 mg/d	8 wk	HAMD; Effective rate;	Self-sweating (1/0) Stomachache (0/1)	1
HuY,2021	China	RCT	DSM-IV	134 (49/68) 62.14%	e.g.:43.69 (12.78) CG:41.37 (14.00)	2 parallel arms (KXS; KXS placebo)	KXS	KXS placebo 4 tablet/d	8 wk	HAMD; SDS;	Adverse events (3/2)	7
Sun,2021	China	RCT	ECDTAD	80 (40/40) 58.75%	e.g.:62.77 (5.34) CG:63.07 (5.97)	2 parallel arms (KXS; AD)	KXS plus Escitalopram Oxalate	Escitalopram Oxalate 10 mg/d	6 wk	SDS;	NR	2
Tan,2016	China	RCT	CCMD-3	60 (36/24) 76.67%	e.g.:65.25 (7.94) CG:67.35 (4.90)	2 parallel arms (KXS; AD)	KXS	Fluoxetine 20 mg/d	8 wk	HAMD; Effective rate;	Anorexia (0/2) Dizziness (0/1)	1
Tang,2019	China	RCT	NA	100 (50/50) 48.00%	e.g.:58.86 (6.54) CG:58.10 (6.33)	2 parallel arms (KXS; AD)	KXS plus Deanxit	Deanxit 21 mg/d	4 wk	HAMD;	Adverse events (3/2)	0
Yu,2020	China	RCT	CCMD-3	78 (39/39) 53.58%	e.g.:65.41 (8.68) CG:64.10 (8.32)	2 parallel arms (KXS plus AD; AD)	KXS	Deanxit 21 mg/d	4 wk	HAMD; Effective rate;	0	3
Zhang,2014	China	RCT	CCMD-3	60 (35/25) 85.00%	e.g.:69.00 (8.50) CG:67.20 (5.07)	2 parallel arms (KXS; AD)	KXS	Citalopram 20 mg/d	8 wk	HAMD; Effective rate;	0	1
Zhu,2010	China	RCT	CCMD-3	60 (30/30) 63.33%	e.g.:43.03 (11.85) CG:44.13 (12.68)	2 parallel arms (KXS; AD)	KXS	Fluoxetine 20 mg/d	6 wk	Effective rate;	Constipation (0/1)	3
Zhu,2018	China	RCT	DSM-IV ICD-10	75 (34/41) 69.33%	e.g.:48.47 (14.41) CG:44.61 (12.13)	2 parallel arms (KXS plus AD placebo; AD plus KXS placebo)	KXS plus Fluoxetine placebo	Fluoxetine 20 mg/d plus KXS placebo 4 tablet/d	8 wk	HAMD; SDS;	Dry mouth (1/1) Disgusting (0/1) Heartburn (0/1) Headache (0/1) Granulopenia (0/1)	7

AD *=* antidepressant, CCMD-3 *=* Chinese Classification and Diagnostic Criteria of Mental Disorders, 3rd Edition, CG *=* control group, DSM-IV *=* Diagnostic and Statistical Manual of Mental Disorders, 4th Edition, ECDTAD *=* Expert consensus on the diagnosis and treatment of anxiety and depression in general hospitals, e.g. *=* experimental group, HAMD *=* Hamilton Depression Scale, ICD-10 *=* International Classification of Diseases 10th edition, KXS *=* Kaixinsan, NA *=* not available, NRNot Reported, RCT *=* randomized controlled trial, SDS *=* Self-Rating Depression Scale.

The main observables were various depression scale scores, mainly the HAMD^[29]^ and the SDS.^[30]^ A score of 0 to 7 on the HAMD scale is average without depression, a score of 8 to 16 indicates mild depression, 17 to 23 indicates moderate depression, and a score of more than 24 indicates severe depression.^[31]^ The SDS consists of 20 questions, each rated on a scale of 1 to 4. After the rating was completed, the scores for each of the 20 items were added to obtain an approximate total. This was then multiplied by 1.25 and rounded to the nearest whole number to obtain the standardized score.^[30]^ A score of 53 to 62 is considered mild depression, 63 to 72 is considered moderate, and > 72 indicates severe depression. The secondary metrics observed were the total number of effectiveness and safety metrics. Clinical response was assessed based on the reduction in HAMD scores for depression as follows: recovery: ≥ 75% reduction; significantly effective: ≥ 50% reduction; improvement: ≥ 25% reduction; and ineffective: < 25% reduction. The total number of effective persons was defined as the total number of subjects with a ≥ 25% reduction in their HAMD scores from baseline to endpoint. The investigators assessed all outcome measures at the end of each treatment session. Safety indicators included the type and number of adverse reactions.

### 2.5. Data extraction and quality assessment

We assessed the methodological quality of each included experiment using the Modified Jadad Scale, which was expanded from the original 3 sections to 4 sections with scores ranging from 0 to 7, with scores of 1 to 3 considered to be of low quality and scores of 4 to 7 deemed to be of high-quality.^[32–34]^ The Cochrane Collaboration tool^[35]^ was used to assess the risk of bias. The tool includes sequence generation; allocation concealment; blinding of participants, personnel, and outcome assessors; incomplete outcome data; selective outcome reporting; and other sources of bias. We categorized subjects into 3 categories: “low risk,” “high risk,” and “unclear risk.” Two authors completed the scoring process, and any discrepancies that arose throughout the assessment process were assessed by a third author. Any discrepancies throughout the evaluation process were reviewed by a third reviewer and resolved by consensus. The Jadad scores for each study were agreed upon and are summarized.

### 2.6. Statistical analysis

If the data recorded in a study could not be used directly in our analysis, they were converted into mean and standard deviation formats.^[36]^ We analyzed and processed the data using the STATA 17 software (StataCorp LLC, College Station, TX, USA). Pooled relative risks (RRs) and 95% confidence intervals (CIs) were calculated for dichotomous variables, and weighted mean differences (WMDs) and 95% confidence intervals were calculated for continuous variables. The chi-square test was used to estimate heterogeneity, and the I^2^ statistic evaluated the heterogeneity between studies and categorized them as 25% (low heterogeneity), 50% (moderate heterogeneity), or 75% (high heterogeneity).^[37]^ If significant heterogeneity was identified, a random effects model was applied to the meta-analysis; otherwise, a fixed effects model was used. We analyzed the data into subgroups, such as the north-south location of the region where the experiment was conducted, treatment duration, experimental design, dosage form, type of antidepressant medication in the control group, and the quality level of the literature. Studies located in China were differentiated based on the Qinling-Huaihe line^[38]^ and the geographic demarcation line between northern and southern China, while the north-south categorization in other countries or regions was assessed separately based on the situation of the country in which they were located. Potential publication bias was evaluated by examining the distribution of funnel plots.

In the presence of publication bias, the effect of publication bias on the pooled results was further investigated using the cut-and-patch method. Sensitivity analyses were conducted to detect the presence of high-impact studies that could distort the results.

## 3. Results

### 3.1. Search results

**Figure 1.**
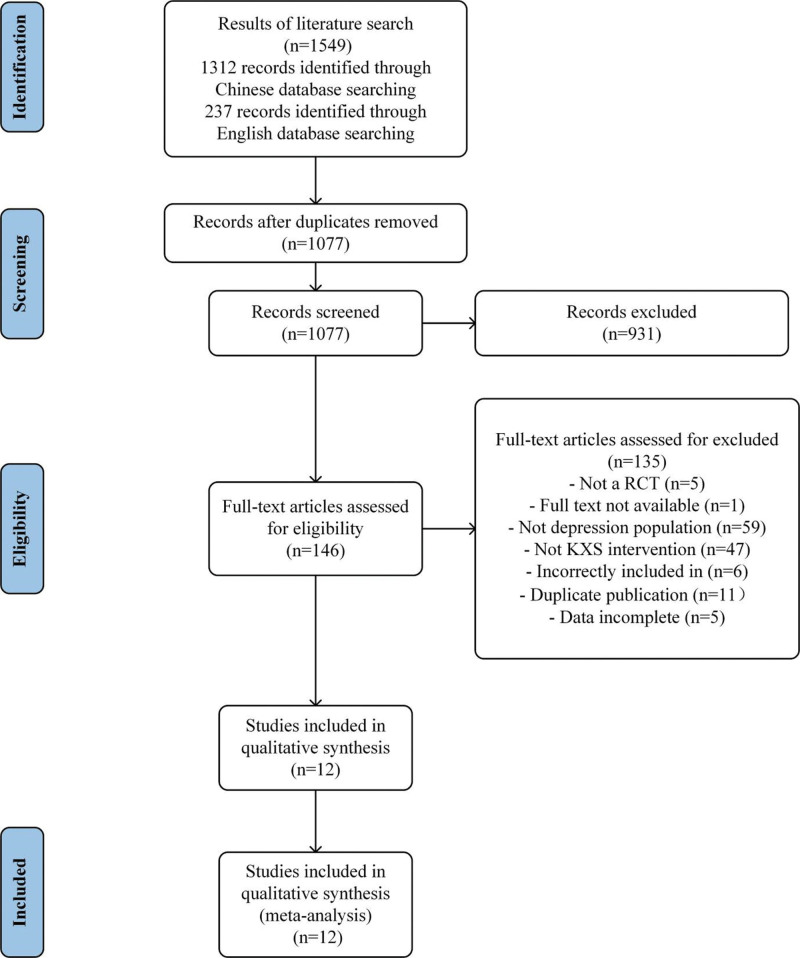
Process of study selection following the Preferred Reporting Items for Systematic Reviews and Meta-Analyses (PRISMA).

A total of 1549 studies were retrieved. There were 1312 studies in Chinese and 237 in English, leaving 1077 records after excluding duplicates. A total of 12 studies were finally included after exclusions (Hu, 2021^[25]^; Bao, 2011^[26]^; Sun, 2021^[27]^; Chen, 2014^[39]^; HuY, 2021^[40]^; Huang, 2019^[41]^; Tan, 2016^[42]^; Tang, 2019^[43]^; Yu, 2020^[44]^; Zhang, 2014^[45]^; Zhu, 2010^[46]^; Zhu, 2018^[47]^). A PRISMA flowchart of the article screening process is shown in Figure 1.

### 3.2. Study characteristics

Table 1 summarizes the characteristics of 12 studies conducted between 2010 and 2021. All eligible studies were conducted in China, were 2-arm trials, and 2 were published in English. The diagnostic criteria used included CCMD-3 (Bao, 2011; Chen, 2014; Tan, 2016; Yu, 2020; Zhang, 2014; Zhu, 2010), DSM-IV (Hu, 2021; HuY, 2021; Zhu, 2018), ICD-10^[41]^ and other (Sun, 2021); only 1 study (Tang, 2019) did not specify the diagnostic criteria. The sample size for inclusion in the study ranged from 70 to 134, and 1034 participants were recruited, including 527 and 507 participants in the treatment and control groups, respectively. The treatment duration ranged from 4 to 8 weeks, with 8 weeks being the most frequent. The main measures used in the control group were antidepressants (Fluoxetine, Citalopram, and Deanxit) and the KXS placebo. Eleven of the studies (Bao, 2011; Chen, 2014; Hu, 2021; HuY, 2021; Huang, 2019; Tan, 2016; Tang, 2019; Yu, 2020; Zhang, 2014; Zhu, 2010; Zhu, 2018) reported side effect profiles, mainly adverse drug reactions, dizziness, gastrointestinal reactions, dry mouth, etc, while the remaining study did not report on side effects.

Table 2 shows the specific applications of Chinese medicines. Chinese medicines are administered in the form of tablets and soups, the majority of which are in the form of soups, with a total of 8 items (Bao, 2011; Chen, 2014; Sun, 2021; Tan, 2016; Tang, 2019; Yu, 2020; Zhang, 2014; Zhu, 2010). All studies used standardized botanical drugs; however, adequate reporting of botanical materials was insufficient, and overall, taxonomically inadequate. In terms of medication details, 5 studies (Bao, 2011; Chen, 2014; Hu, 2021; HuY, 2021; Zhu, 2018) followed the original formula composition, while others were modified, with 1 study^[44]^ replacing the main herb Renshen (*Panax ginseng* C. A. Mey.) with Dangshen (*Codonopsis pilosula* (Franch.) Nannf.). However, this change was not considered in this study.

**Table 2:** Major herbal components of KXS decoction.

Study ID	Dosage type	Botanical drug selection	Dose and period	Adequate reporting on botanical materials	Major herbal components
Bao,2011	Decoction	Standardized	1 dose/d for 6 wk	C	Renshen (*Panax ginseng* C. A. Mey.), Fuling (*Poria cocos* (Schw.) Wolf), Yuanzhi (*Polygala tenuifolia* Willd.), Shichangpu (*Acorus tatarinowii* Schott)
Chen,2014	Decoction	Standardized	KXS 1 dose/d + Fluoxetine 20 mg/d for 8 wk	C	Renshen (*Panax ginseng* C. A. Mey.), Fuling (*Poria cocos* (Schw.) Wolf), Yuanzhi (*Polygala tenuifolia* Willd.), Shichangpu (*Acorus tatarinowii* Schott)
Hu,2021	Tablet	Standardized	KXS 3.2 g/d + AD placebo 20 mg/d for 8 wk	C	Renshen (*Panax ginseng* C. A. Mey.), Fuling (*Poria cocos* (Schw.) Wolf), Yuanzhi (*Polygala tenuifolia* Willd.), Shichangpu (*Acorus tatarinowii* Schott)
Huang,2019	Tablet	Standardized	9 g/d for 8 wk	C	Renshen (*Panax ginseng* C. A. Mey.), Fuling (*Poria cocos* (Schw.) Wolf), Yuanzhi (*Polygala tenuifolia* Willd.), Chaihu (*Bupleurum chinense* DC.), Bajitian (*Morinda officinalis* How), Chishao (*Paeonia veitchii* Lynch), Zhishi (*Fructus Aurantii* Immaturus), Gancao (*Glycyrrhiza uralensis* Fisch.)
HuY,2021	Tablet	Standardized	4 tablet/d for 8 wk	C	Renshen *(Panax ginseng* C. A. Mey.), Fuling *(Poria cocos* (Schw.) Wolf), Yuanzhi (*Polygala tenuifolia* Willd.), Shichangpu (*Acorus tatarinowii* Schott)
Sun,2021	Decoction	Standardized	KXS 1 dose/d + Escitalopram Oxalate 10 mg/d for 6 wk	C	Renshen (*Panax ginseng* C. A. Mey.), Fuling (*Poria cocos* (Schw.) Wolf), Yuanzhi (*Polygala tenuifolia* Willd.), Shichangpu (*Acorus tatarinowii* Schott), Chaihu *(Bupleurum chinense* DC.), Danggui (*Angelica sinensis* (Oliv.) Diels), Baizhu (*Atractylodes macrocephala* Koidz.), Baishao (*Paeonia obovata* var. *glabra* Makino), Chuanxiong (*Ligusticum chuanxiong* Hort.), Taoren (*Persicae* semen), Shudihuang (*Rehmanniae Radix* Praeparata), Baihe (*Lilii* Bulbus), Suanzaoren (*Ziziphus jujuba* var. *spinosa* (Bunge) Hu ex H.F.Chow), Hehuanpi (*Acacia julibrissin* (Durazz.) Willd.), Honghua (*Carthamus tinctorius* Linn.), Huanglian (*Coptis chinensis* Franch.), Zhigancao (*Glycyrrhiza uralensis* Fisch.)
Tan,2016	Decoction	Standardized	1 dose/d for 8 wk	C	Renshen (*Panax ginseng* C. A. Mey.), Fuling (*Poria cocos* (Schw.) Wolf), Yuanzhi (*Polygala tenuifolia* Willd.), Chaihu (*Bupleurum chinense* DC.), Bajitian (*Morinda officinalis* How), Chishao (*Paeonia veitchii* Lynch), Zhishi (*Fructus Aurantii* Immaturus), Gancao (*Glycyrrhiza uralensis* Fisch.)
Tang,2019	Decoction	Individualized	KXS 1 dose/d + Deanxit 21 mg/d for 4 wk	C	Renshen (*Panax ginseng* C. A. Mey.), Fuling (*Poria cocos* (Schw.) Wolf), Yuanzhi (*Polygala tenuifolia* Willd.), Shichangpu (*Acorus tatarinowii* Schott), Zexie (*Alisma plantago-aquatica* Linn.), Baizhu (*Atractylodes macrocephala* Koidz.), Danshen (*Salvia miltiorrhiza* Bunge), Yujin (*Curcuma aromatica* Salisb.), Banxia (*Pinellia ternata* (Thunb.) Makino), Chenpi (*Citrus reticulata* Blanco), Zhishi (*Fructus Aurantii* Immaturus), Chuanxiong (*Ligusticum chuanxiong* Hort.), Zhigancao (*Glycyrrhiza uralensis* Fisch.), Suanzaoren (*Ziziphus jujuba* var. *spinosa* (Bunge) Hu ex H.F.Chow), Dangshen (*Codonopsis pilosula* (Franch.) Nannf.), Huangqi *(Astragalus propinquus* Schischk.), Fushen (*Poria cocos* (Schw.) Wolf), Guiyuan (*Dimocarpus longan* Lour.), Danggui (*Angelica sinensis* (Oliv.) Diels)
Yu,2020	Decoction	Standardized	KXS 1 dose/d + Deanxit 21 mg/d for 4 wk	C	Dangshen (*Codonopsis pilosula* (Franch.) Nannf.), Fuling (*Poria cocos* (Schw.) Wolf), Yuanzhi (*Polygala tenuifolia* Willd.), Shichangpu (*Acorus tatarinowii* Schott), Huangqi (*Astragalus propinquus* Schischk.), Danshen (*Salvia miltiorrhiza* Bunge), Chuanxiong (*Ligusticum chuanxiong* Hort.), Guizhi (*Cinnamomi* Ramulus), Jiangxiang (*Dalbergia odorifera* T. Chen), Xiangfu (*Cyperi* Rhizoma), Sanqi (*Panax notoginseng* (Burkill) F. H. Chen ex C. H. Chow), Huanglian (*Coptis chinensis* Franch.)
Zhang,2014	Decoction	Standardized	1 dose/d for 8 wk	C	Renshen (*Panax ginseng* C. A. Mey.), Fuling (*Poria cocos* (Schw.) Wolf), Yuanzhi (*Polygala tenuifolia* Willd.), Chaihu (*Bupleurum chinense* DC.)Bajitian (*Morinda officinalis* How), Chishao (*Paeonia veitchii* Lynch), Zhishi (*Aurantii FructusImmaturus*), Gancao (*Glycyrrhiza uralensis* Fisch.)
Zhu,2010	Decoction	Standardized	1 dose/d for 6 wk	C	Renshen (*Panax ginseng* C. A. Mey.), Fuling (*Poria cocos* (Schw.) Wolf), Yuanzhi (*Polygala tenuifolia* Willd.), Shichangpu (*Acorus tatarinowii* Schott), Longchi (*Dens* Draconis), Baishao *(Paeonia obovata* var. *glabra* Makino), Baizhu (*Atractylodes macrocephala* Koidz.), Fushen (*Poria cocos* (Schw.) Wolf), Danggui (*Angelica sinensis* (Oliv.) Diels)
Zhu,2018	Tablet	Standardized	KXS 3.2 g/d + AD placebo 20 mg/d for 8 wk	C	Renshen (*Panax ginseng* C. A. Mey.), Fuling (*Poria cocos* (Schw.) Wolf), Yuanzhi (*Polygala tenuifolia* Willd.), Shichangpu (*Acorus tatarinowii* Schott)

The reporting was rated by 3 levels: A, full information about the botanical material is provided including a voucher specimen; B, only partial information about the botanical material is provided but a voucher specimen is missing and there are taxonomic inaccuracies; C, inadequate information and overall taxonomically inadequate.

### 3.3. Study quality

**Figure 2.**
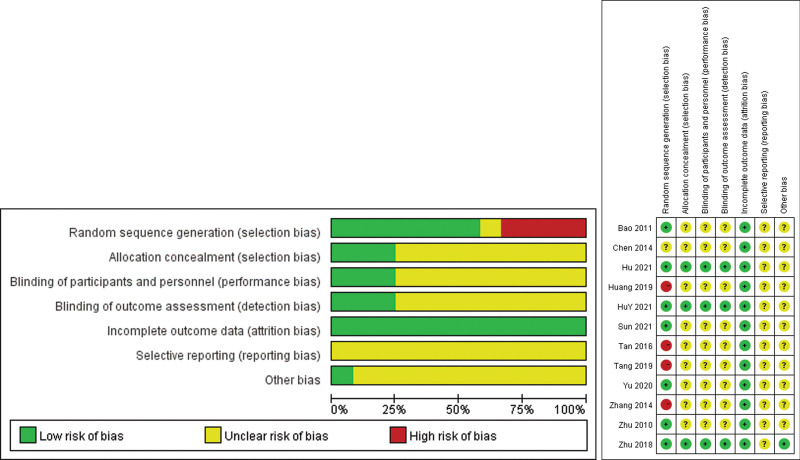
Risk of Bias graph and summary.

Table 1 presents the methodological qualities of the included studies. Overall, the quality of the studies ranged from fair to good, with 41.7% categorized as good and 58.3% as poor. Three studies (Hu, 2021; HuY, 2021; Zhu, 2018) used a hidden allocation that was more standardized, including concealment by the investigator, subjects, and drug dispenser, and all studies reported random allocation. All included studies were blinded to the participants, and all outcome data were processed. Only 1 study (Zhu, 2018) has reported other sources of bias in detail. The risk of bias assessment for all the included studies is shown in Figure 2.

### 3.4. Synthetic results

**Figure 3.**
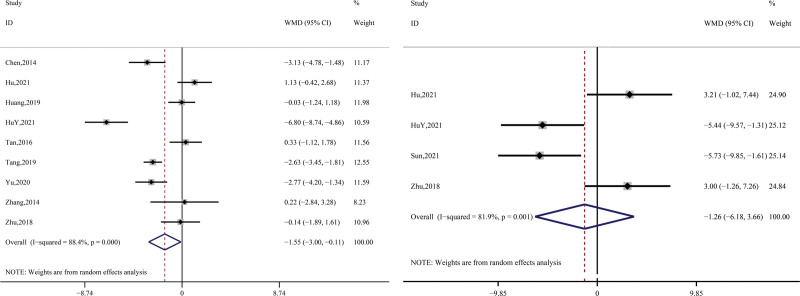
Synthetic result for HAMD and SDS. HAMD = Hamilton Rating Scale for Depression, SDS = Self-Rating Depression Scale.

Regarding the evaluation of depressed mood, 9 studies (Chen, 2014; Hu, 2021; HuY, 2021; Huang, 2019; Tan, 2016; Tang, 2019; Yu, 2020; Zhang, 2014; Zhu, 2018) were conducted using the Hamilton Depression Scale, and the data synthesized from each study had large heterogeneity (*P* < .01, I^2^ = 88.4%), so they were analyzed in a random manner. The results showed that compared to the control group, the treatment groups all showed greater improvement (WMD = −1.55, 95% CI = -3.00~-0.11, *z* = 2.11, *P* = .04); a total of 4 studies (Hu, 2021; HuY, 2021; Sun, 2021; Zhu, 2018) documented in detail the SDS scale, with similarly large post-synthesis heterogeneity (*P* < .01, I^2^ = 81.9%), which was also analyzed using a random-effects model. The results showed a non-significant difference between the control and treatment groups in terms of overall improvement (WMD = −1.26, 95% CI = −6.18 3.66, *z* = 0.50, *P* = .62). Further details are shown in Figure 3.

**Figure 4.**
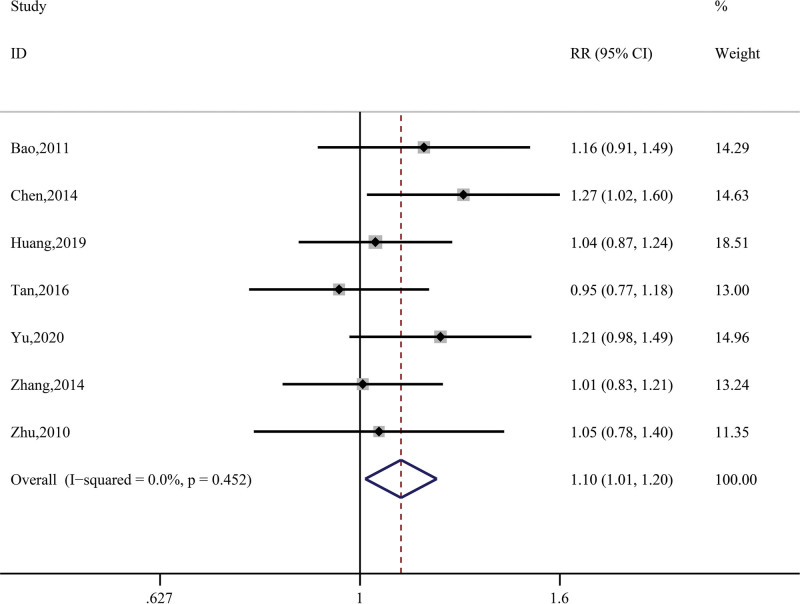
Synthetic result for effectiveness.

A total of 8 studies in this research (Bao, 2011; Chen, 2014; Huang, 2019; Sun, 2021; Tan, 2016; Yu, 2020; Zhang, 2014; Zhu, 2010) recorded effectiveness by counting the number of people with effective treatment. The data were synthesized using a fixed-effects model after a heterogeneity test (*P* = .45, I^2^ = 0%), which showed that the overall effective rate of the treatment group was due to the control group. The data differed significantly (RR = 1.10, 95% CI = 1.01 ~ 1.20, *z* = 2.25, *P* = .03); see Figure 4.

**Figure 5.**
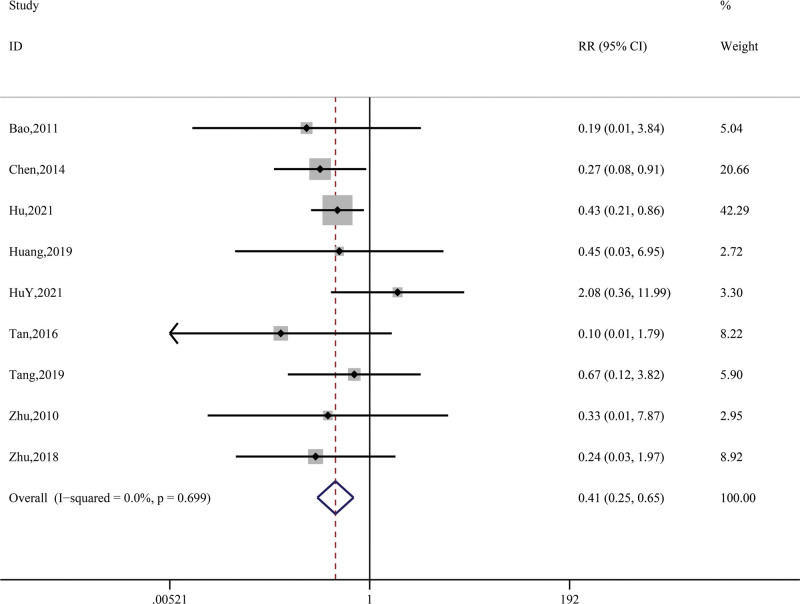
Synthetic result for side effects.

Finally, 11 studies (Bao, 2011; Chen, 2014; Hu, 2021; HuY, 2021; Huang, 2019; Tan, 2016; Tang, 2019; Yu, 2020; Zhang, 2014; Zhu, 2010; Zhu, 2018) were conducted for the occurrence of side effects, which mainly included adverse drug reactions, dry mouth, and gastrointestinal discomfort (see Table 1). After the test of heterogeneity (*P* = .70, I^2^ = 0.0%) and analysis using the fixed-effect model, the results showed that the frequency of side effects in the control group was higher than that in the treatment group, and there was a statistically significant difference (RR = 0.41, 95% CI = 0.25~ 0.65, *z* = 3.71, *P* < .01). See Figure 5.

**Figure 6.**
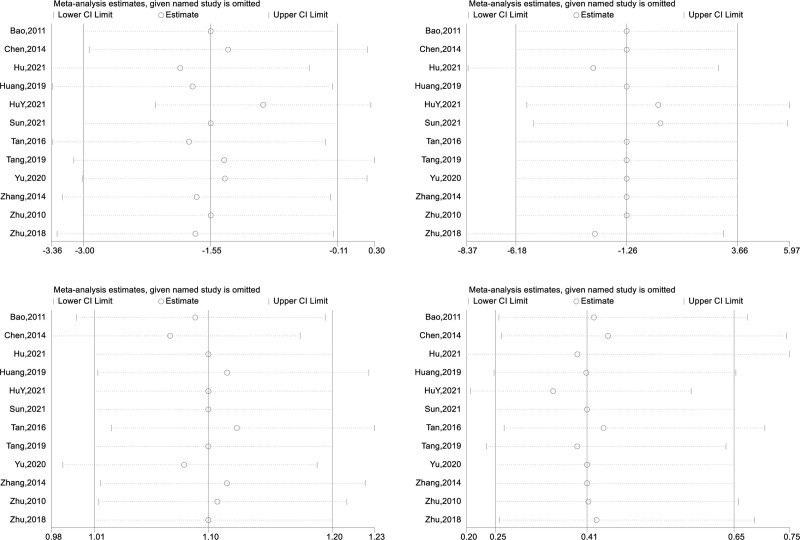
Result of sensitivity analysis.

According to the sensitivity analysis (Fig. 6), no study significantly influenced the final results, regardless of HAMD, SDS, treatment effectiveness, or the occurrence of side effects. No study was considered nonsignificant because its removal had no significant effect on the overall effect (i.e., a change from significant to nonsignificant). Although there was considerable heterogeneity across studies in some of the scales, it could be reduced with moderators such as the geographic region in the north and south of the country, the duration of treatment, and the control group setting.

### 3.5. Moderator analysis

**Figure 7.**
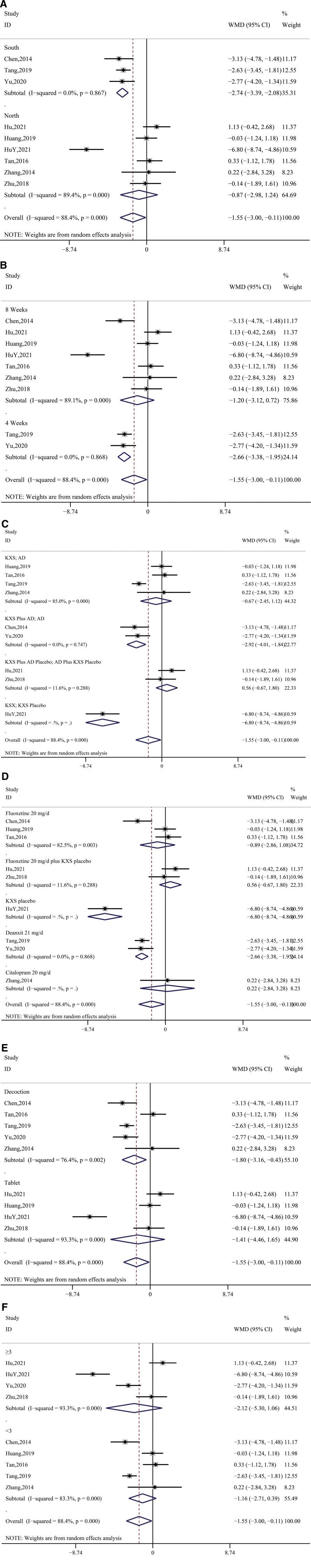
Moderator analysis for HAMD. (A) Region; (B) Period; (C) Design; (D) Control group; (E) Dosage type; (F) Study quality. HAMD = Hamilton Rating Scale for Depression.

**Figure 8.**
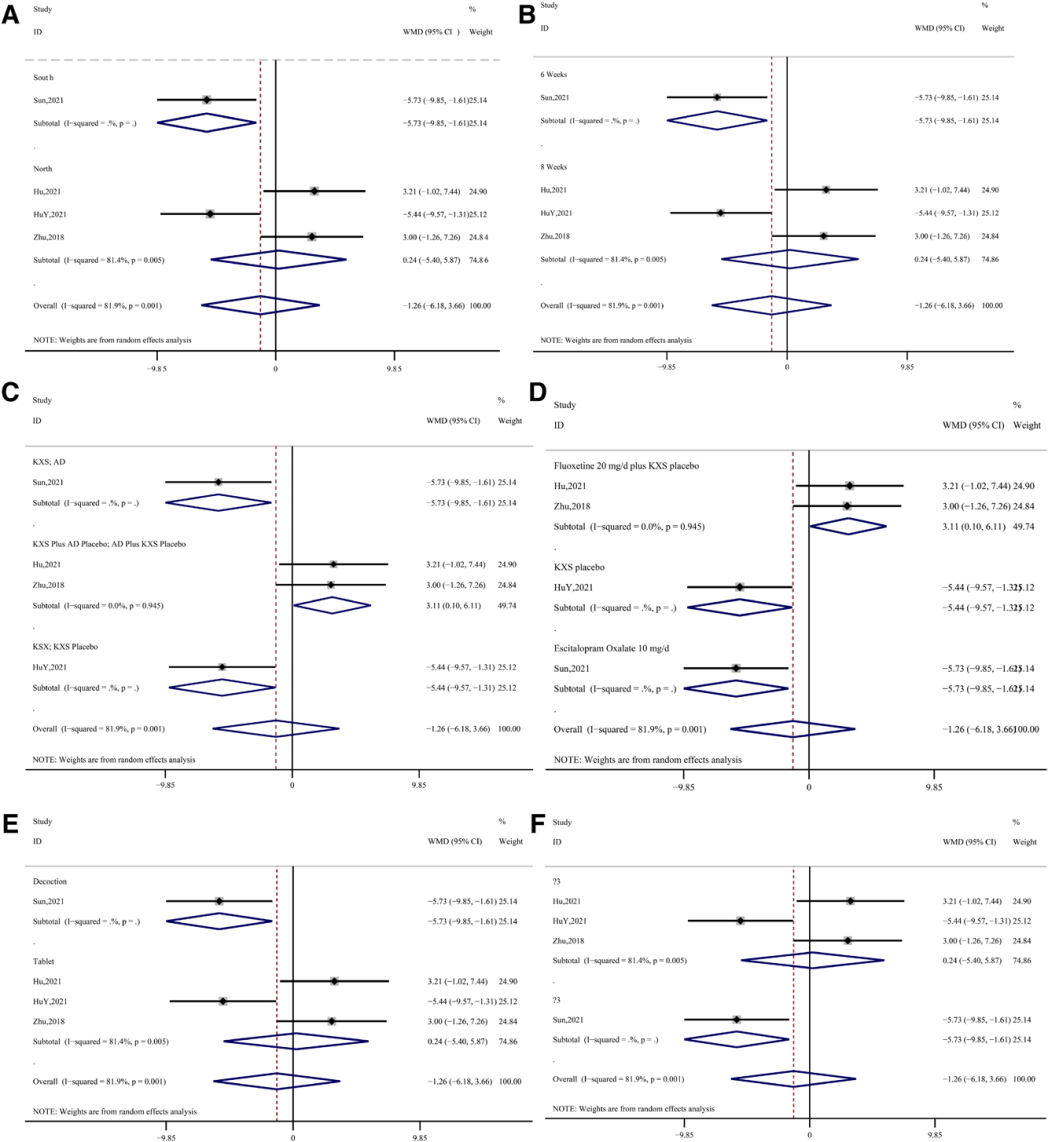
Moderator analysis for SDS. (A) Region; (B) Period; (C) Design; (D) Control group; (E) Dosage type; (F) Study quality. SDS = Self-Rating Depression Scale.

The dichotomous and continuous variables counted in this study were analyzed using the factors in the Table 1 for moderation. Subgroup analysis based on the HAMD results showed that the KXS group was more effective in terms of patient location in the South (*P* = .87, I^2^ = 0.0%; 95%CI = -3.39~-2.08, *P* < .01) and 4 weeks of treatment (*P* = .87, I^2^ = 0.0%; 95%CI = -3.38~-1.95, *P* < .01), with better results in KXS + AD as well as the experimental design of AD, the experimental design of KXS versus the KXS placebo, the use of Deanxit as well as the KXS placebo for the control western medicine, and the use of the soup dosage form for KXS were significant in terms of the experimental design of AD, the experimental design of KXS versus KXS placebo, the use of Deanxit as well as the KXS placebo for the control Western medicine, and the use of the soup dosage form for KXS (*P* < .05). The SDS scale score was better regarding the patient location in the south, treatment for 8 weeks, and use of a soup dosage form for the KXS group (see Figs. 7 and 8 and Tables 3 and 4).

**Table 3:** HAMD subgroup analysis results.

Variables	Heterogeneity		Effect size				
	*P* value	I-squared	WMD	95% CI		*z*-value	*P* value
Overall	<.001	88.4%	-1.56	-3.00	-0.11	2.11	.035
Region							
South	.867	0.0%	-2.74	-3.39	-2.08	8.19	<.001
North	<.001	89.4%	-0.87	-2.98	1.24	0.80	.421
Period							
8 wk	<.001	89.1%	-1.20	-3.12	0.72	1.22	.222
4 wk	.868	0.0%	-2.67	-3.38	-1.95	7.31	<.001
Design							
KXS vs AD	<.001	87.8%	-0.68	-2.45	1.12	0.73	.465
KXS + AD vs AD	.963	0.0%	-2.93	-4.01	-1.84	5.30	<.001
KXS + AD placebo vsAD + KXS Placebo	.324	0.0%	0.57	-0.67	1.80	0.89	.371
KXS vs KXS placebo	-	-	-6.80	-8.74	-4.86	6.88	<.001
Control Group Medicine							
Fluoxetine	.003	82.5%	-0.89	-2.86	1.08	0.88	.377
Fluoxetine + KXS Placebo	.288	11.6%	0.57	-0.67	1.80	0.89	.371
Deanxit	.868	0.0%	-2.67	-3.38	-1.95	7.31	<.001
KXS placebo	-	-	-6.80	-8.74	-4.86	6.88	<.001
Citalopram	-	-	0.22	-2.85	3.29	0.14	.035
Dosage Type							
Decoction	.002	76.4%	-1.80	-3.16	-0.43	2.58	.010
Tablet	<.001	93.3%	-1.41	-4.46	1.65	0.90	.366
Study Quality (Based on Jadad Score)							
>=3	<.001	93.3%	-2.116	-5.297	1.065	1.3	.192
<3	<.001	83.3%	-1.164	-2.713	0.385	1.47	.141

**Table 4:** SDS subgroup analysis results.

Variables	Heterogeneity		Effect size				
	*P* value	I-squared	WMD	95% CI		*z*-value	*P* value
Overall	.001	81.9%	-1.26	-6.18	3.66	0.50	.615
Region							
South	-	-	-5.73	-9.85	-1.61	2.73	.006
North	.005	81.4%	0.24	-5.40	5.87	0.08	.934
Period							
8 wk	.005	81.4%	0.24	-5.40	5.87	0.08	.934
6 wk	-	-	-5.73	-9.85	-1.61	2.73	.006
Design							
KXS vs AD	-	-	-5.73	-9.85	-1.61	2.73	.006
KXS + AD placebo vs AD + KXS Placebo	.945	0.0%	3.11	0.10	6.11	2.03	.043
KXS vs KXS placebo	-	-	-5.44	-9.57	-1.31	2.58	.010
Control Group Medicine							
Fluoxetine + KXS Placebo	.945	0.0%	3.11	0.10	6.11	2.03	.043
KXS placebo	-	-	-5.44	-9.57	-1.31	2.58	.010
Escitalopram Oxalate	-	-	-5.73	-9.85	-1.61	2.73	.006
Dosage Type							
Decoction	-	-	-5.73	-9.85	-1.61	2.73	.006
Tablet	.005	81.4%	0.24	-5.40	5.87	0.08	.934
Study Quality (Based on Jadad Score)							
>=3	.005	81.4%	0.24	-5.40	5.87	0.08	.934
<3	-	-	-5.73	-9.85	-1.61	2.73	.006

**Figure 9.**
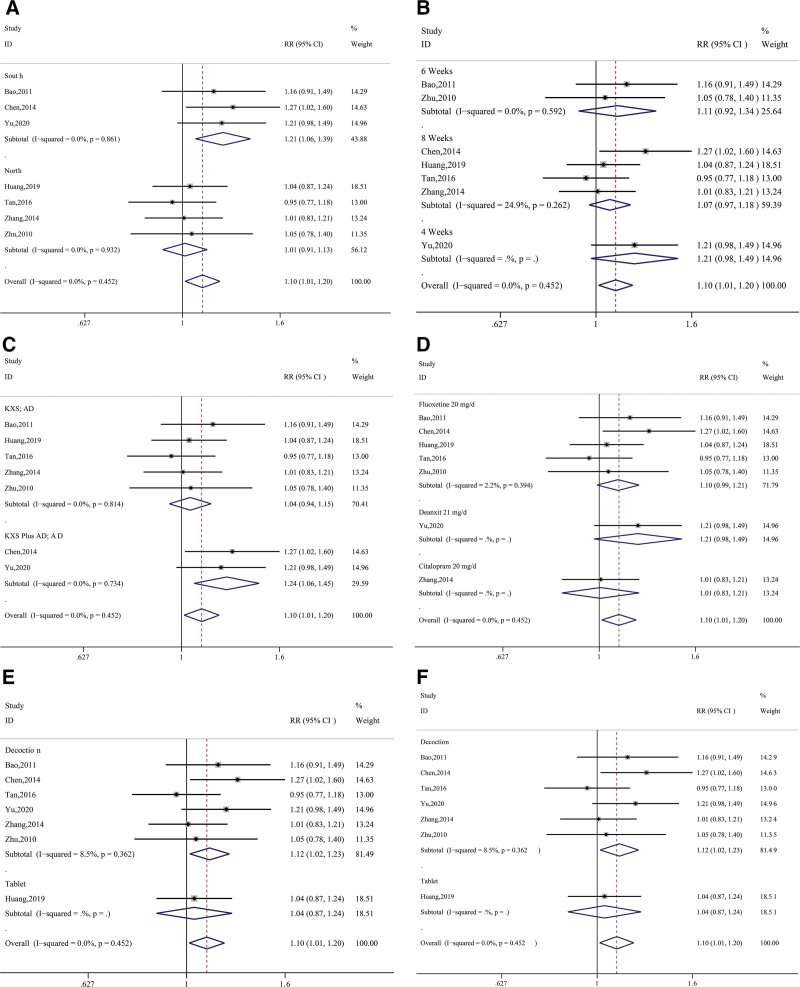
Moderator analysis for effective situation. (A) Region; (B) Period; (C) Design; (D) Control group; (E) Dosage type; (F) Study quality.

**Figure 10.**
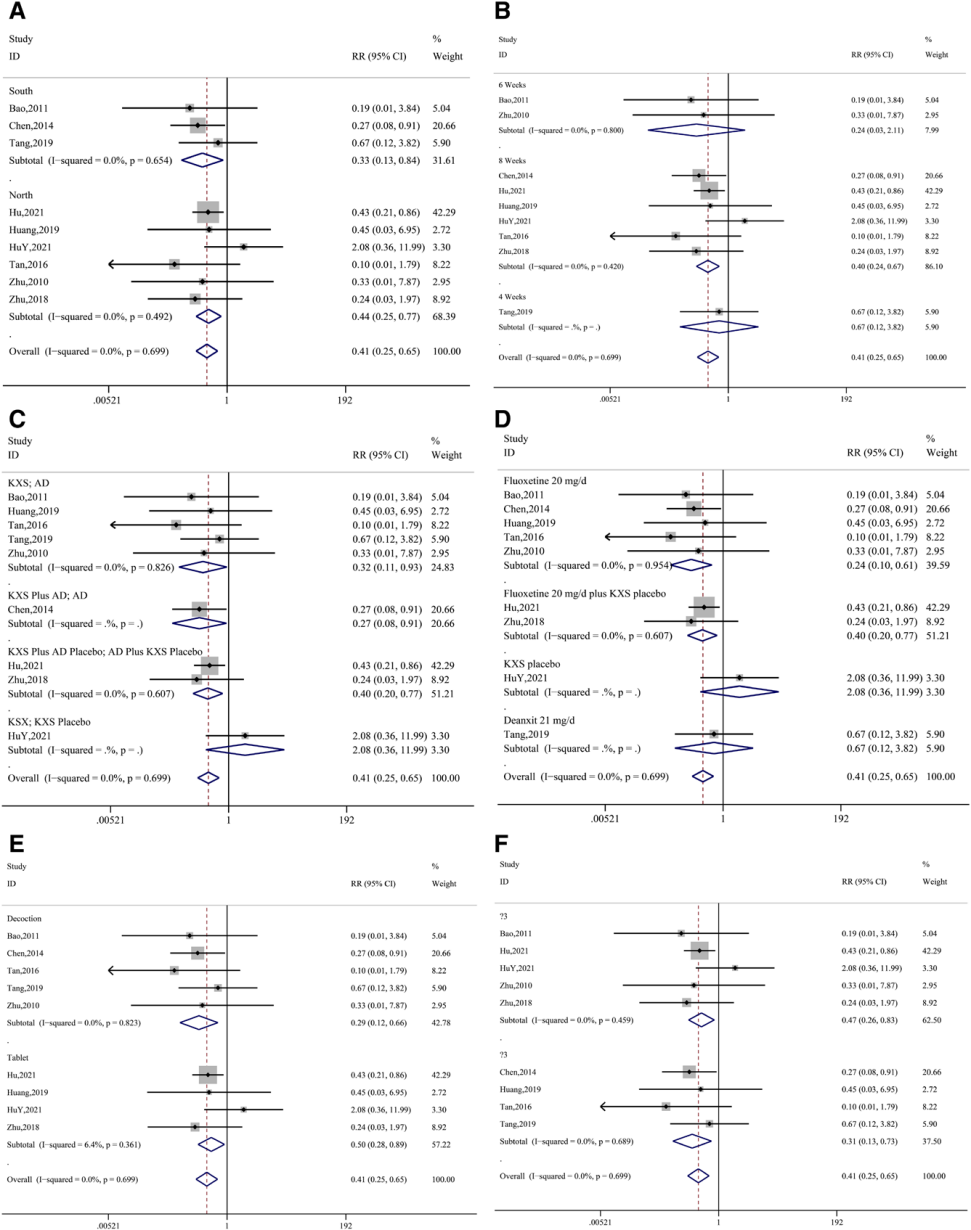
Moderator analysis for side effects. (A) Region; (B) Period; (C) Design; (D) Control group; (E) Dosage type; (F) Study quality.

In terms of efficacy, the results of subgroup analysis showed that the KXS group had a better outcome when the patients were in the southern region of China (*P* = .86, I^2^ = 0.0%; 95%CI = 1.06~1.39, *P* < .01), the experimental design was KXS plus AD and AD (*P* = .73, I^2^ = 0.0%, 95%CI = 1.06~1.45, *P* < .01) and the dosage form of KXS was soup (*P* = .36, I^2^ = 8.5%; 95%CI = 1.02~1.23, *P* < .01); these variables showed better efficacy (Fig. 9 and Table 5). The results of various subgroup analyses showed a lower occurrence of side effects in the KXS group, which was particularly significant in the 3 categories of patient location, duration of treatment, and dosage form (*P* < .05). In addition, the quality grading of the literature, experimental design, and type of antidepressant were significant as shown in Figure 10 and Table 6.

**Table 5:** Effective situation subgroup analysis results.

Variables	Heterogeneity		Effect size				
	*P* value	I-squared	RR	95% CI		*z*-value	*P* value
Overall	.452	0.0%	1.10	1.01	1.20	2.25	.025
Region							
South	.861	0.0%	1.21	1.06	1.39	2.88	.004
North	.932	0.0%	1.01	0.91	1.13	0.22	.829
Period							
8 wk	.262	24.9%	1.07	0.97	1.18	1.31	.191
6 wk	.592	0.0%	1.11	0.92	1.34	1.08	.280
4 wk	-	-	1.03	0.98	1.49	1.73	.083
Design							
KXS vs AD	.814	0.0%	1.04	0.94	1.15	0.82	.415
KXS + AD vs AD	.734	0.0%	1.24	1.06	1.45	2.72	.007
Control Group Medicine							
Fluoxetine	.394	2.2%	1.10	0.99	1.21	1.76	.078
Deanxit	-	-	1.21	0.98	1.49	1.73	.083
Citalopram	-	-	1.01	0.84	1.21	0.07	.946
Dosage Type							
Decoction	.362	8.5%	1.12	1.02	1.23	2.27	.023
Tablet	-	-	1.04	0.87	1.24	0.39	.693
Study Quality (Based on Jadad Score)							
>=3	.734	0.0%	1.15	0.99	1.31	1.87	.061
<3	.262	24.9%	1.07	0.97	1.18	1.31	.191

**Table 6:** Side effect subgroup analysis results.

Variables	Heterogeneity		Effect size				
*P* value	I-squared	RR	95% CI		*z*-value	*P* value
Overall	.70	0.0%	0.41	0.25	0.65	3.71	<.001
Region							
South	.65	0.0%	0.33	0.13	0.84	2.33	.020
North	.49	0.0%	0.44	0.25	0.77	2.90	.004
Period							
8 wk	.42	0.0%	0.40	0.24	0.67	3.49	<.001
6 wk	.80	0.0%	0.24	0.03	2.12	1.28	.200
4 wk	-	-	0.67	0.12	3.82	0.46	.649
Design							
KXS vs AD	.83	0.0%	0.32	0.11	0.93	2.10	.036
KXS + AD vs AD	-	-	0.27	0.08	0.91	2.11	.035
KXS + AD placebo vs AD + KXS Placebo	.61	0.0%	0.40	0.20	0.77	2.75	.006
KXS vs KXS placebo	-	-	2.08	0.36	11.99	0.82	.412
Control Group Medicine							
Fluoxetine	.95	0.0%	0.24	0.10	0.61	3.01	.003
Fluoxetine + KXS Placebo	.67	0.0%	0.40	0.20	0.77	2.75	.006
Deanxit	-	-	0.67	0.12	3.82	0.46	.649
KXS placebo	-	-	2.08	0.36	11.99	0.82	.412
Dosage Type							
Decoction	.82	0.0%	0.29	0.12	0.66	2.92	.004
Tablet	.36	6.4%	0.50	0.28	0.89	2.37	.018
Study Quality							
>=3	.46	0.0%	0.47	0.26	0.83	2.62	.009
<3	.69	0.0%	0.31	0.13	0.73	2.68	.007

### 3.6. Meta-regression

The results showed that regression analysis based on the perspective of location, treatment duration, control design scheme, and control measures explained 33.6% of HAMD heterogeneity (from tau^2^ = 4.14 to tau^2^ = 2.75). The SDS scale showed a more significant change in heterogeneity after the inclusion of the north-south perspective of the region (from tau^2^ = 20.62 to tau^2^ = 19.91). Regression analyses were not performed for effectiveness and side effects because heterogeneity was not reported.

### 3.7. Publication bias

**Figure 11.**
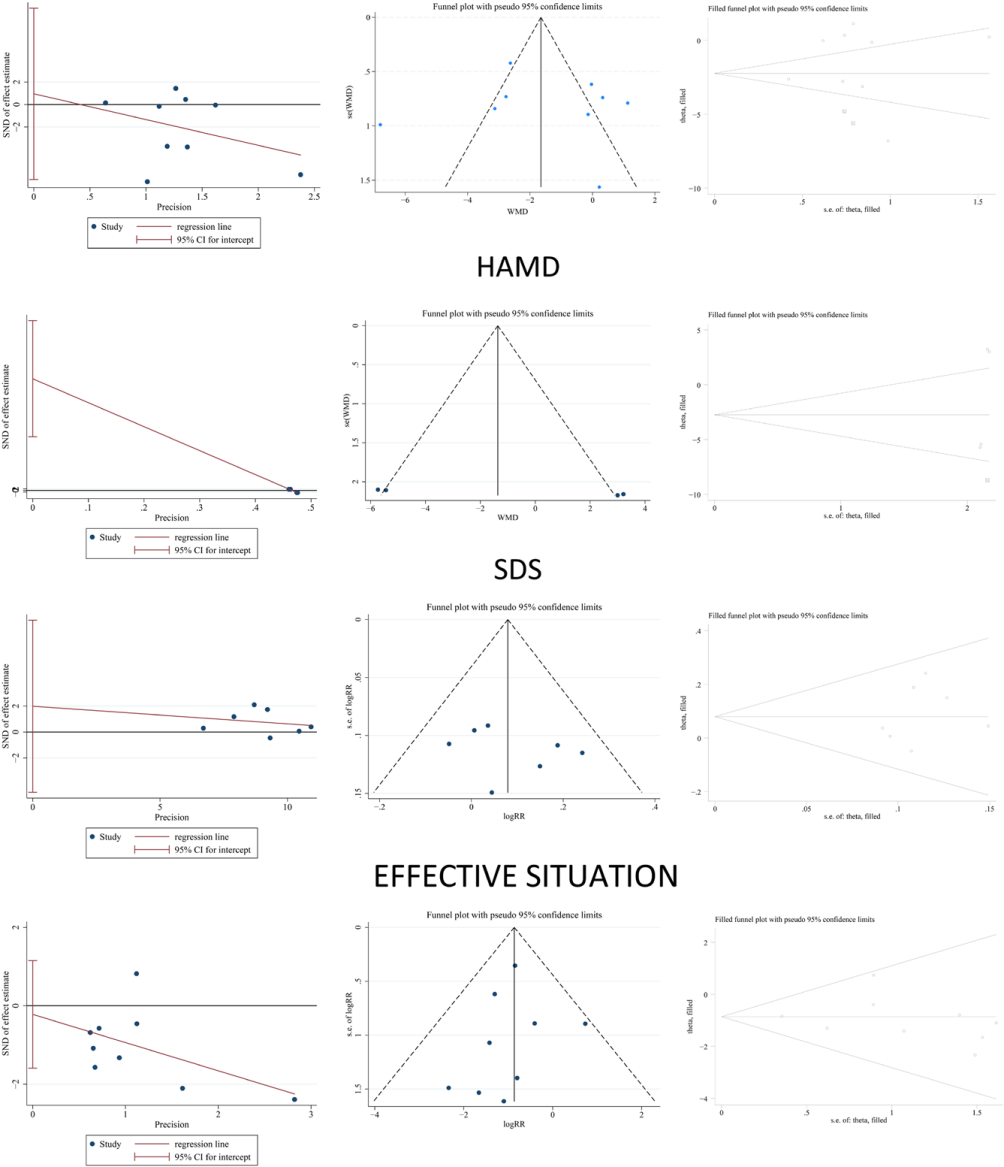
Egger test, funnel plot and the result of trim and fill method.

Egger test and funnel plots were used to assess the publication bias. The asymmetric distribution of the included studies in the funnel plot suggested a publication bias for each dataset, as shown in Figure 11. After testing, there was no publication bias in Hamilton (*P* = .78, 95% CI = −6.67~8.58), effective situation (*P* = .48, 95% CI = -4.63~8.60), and occurrence of side effects (*P* = .72, 95% CI = −1.59~1.15), and to further test the asymmetry in the funnel plots, the clipping method was used to perform calibrations. The results showed that there was a certain degree of publication bias, but the original results remained robust (HAMD’ s 95% CI before: -3.00 ~ −0.11; after 2 items were added: -3.67 ~ −0.80; SDS’ s 95% CI before: −6.18~3.66, after 1 item was added: -7.51~-2.03) after the trim-and-fill method.^[48]^ There was no publication bias in the effectiveness profile (95% CI before: −0.01~0.16; after 0 item added: −0.01~0.16) and the occurrence of side effects (95% CI before: −1.36~-0.37; after 0 item added: −1.36~-0.37). Because studies with small effect sizes were excluded from this meta-analysis, there may have been some cases of bias.

## 4. Discussion

KXS was found to be superior to the control group in terms of HAMD and total effectiveness rate, whereas the SDS difference was nonsignificant. This indicates that KXS is effective in the treatment of depression with antidepressants and that KXS performed better in terms of improvement in HAMD and the total effectiveness rate. In addition, according to the results, KXS had a lower incidence of side effects compared to antidepressants. KXS has also been shown to have a significant therapeutic effect on depression and has a high safety profile in a rat model of depression.^[49]^

The subgroup analysis showed that the effectiveness of KXS in treating depression was related to the region, treatment duration, experimental design, antidepressant control, and dosage form. Among them, the southern region of China and the type of dosage showed significant performance in HAMD, SDS, treatment efficiency, and occurrence of side effects, suggesting that both may be important factors affecting the effectiveness of KXS in treating depression.

In traditional Chinese medicine, there is a concept of “adapting to local conditions,” which refers to medication that may vary slightly for patients in different living areas, leading to different therapeutic effects of the same prescription among different groups. This theory emphasizes the use of local herbal medicines to achieve improved therapeutic effects.

The KXS originated during the Northern Zhou Dynasty and was collected in the “Collection of Inspection Formulas.” The author, Yao Seng Yuan, was born in Huzhou City, Zhejiang Province, China, now located in the southern region of China. Although he had traveled to Xi’an, Shaanxi Province (the northern part of China) as an imperial physician for 3 years, he was forced to move around with the war, and there was a longer period of life in the southern region of China.^[50]^ In addition, KXS is a classic formula recorded and used by Sun Simiao, a Chinese physician from the Tang Dynasty, who, according to historical records, worked in the southern region of China, Sichuan Province, at the time of recording this formula, and carried out diagnosis and treatment.^[51]^ Overall, the KXS-like formulas originated in the southern region of China and were likely used to treat people living there. The main ingredient in KXS is Renshen (*Panax ginseng* C. A. Mey.), Fuling (*Poria cocos* (Schw.) Wolf), Yuanzhi (*Polygala tenuifolia* Willd.), and Shichangpu (*Acorus tatarinowii* Schott.) Among the specific ingredients in KXS, Renseng and Yuanzhi are native to northern China, whereas Fuling and Shichangpu are native to southern China.^[52–55]^ *The climate in southern China is warm and humid all year round, with more precipitation and air humidity than in northern China. Excessive humidity in the external environment is a disease-causing factor in traditional Chinese medicine, and therefore, people in the south often suffer from illnesses due to dampness or abnormal water metabolism.*^[56]^ *In addition, the constitution of people located in the south is mostly phlegmatic-dampness,*^[57]^ *and medicines that regulate the spleen and remove phlegm-dampness can often achieve better results.* Renseng,^[58]^ Fuling,^[59]^ Yuanzhi and Shichangpu^[60]^ have been documented to enhance the function of the spleen (an important organ for regulating the circulation and metabolism of fluid in traditional Chinese medicine) or to directly improve the metabolism of fluid and phlegm. They have antidepressant effects,^[61]^ suggesting that KXS is more suitable for depressed people located in the south. In addition, several studies of traditional herbal tonics worldwide have shown that tablets are slightly less therapeutically effective than traditional decoctions.^[62,63]^ Other types of drugs, such as tablets, are processed through the extraction of native herbal broths and require the addition of excipients in their process, which may be one of the reasons for the difference in effect between different dosage forms.^[64]^ *A portion of the included study on treatment with tablets was produced by compression of dried plant powders, with no other treatment. The original dosage form of KXS was supposed to be a powder, and studies*^[65,66]^ *showed that prescriptions whose original dosage form was a powder showed some reduction in therapeutic efficacy after being changed to a decoction. Still, there was no significant difference in their chemical composition*^[67]^, *precisely the opposite of our analysis. The difference in effect due to dosage form is worthy of further investigation.*

Our meta-analytic study strictly followed the PRISMA specifications, registered the research methodology, used appropriate assessment effect sizes to control for sample size bias, explored possible confounders, and examined the effects of covariates using a regression analysis. These strengths enhance the comprehensiveness and generalizability of the findings. However, the overall methodological quality of the included studies was poor, the sample sizes were small, and the robustness of the findings was questionable. Most RCTs did not describe the randomization and allocation concealment procedures. Therefore, it was impossible to assess whether the randomization process was executed correctly and whether the allocation was adequately concealed. In addition, most studies did not document side effects, and safety evaluations need to be more comprehensive. All controls were other drugs, and the effects of different medications, such as placebo, were not considered.^[68]^ Standardized and individual-based KXS is common in clinical practice. However, we did not compare them because of differences in the research methodology. In the future, we need to find a way to verify which of the 2 is better.

Some aspects of the literature screening process used in this study could be improved. This study only included Chinese and English databases, which may have omitted studies in other languages. Other sources of evidence, such as registries, were not searched, which may have omitted relevant studies, and the screening and extraction of the data were only performed by 2 researchers without cross-checking. These limitations may have affected the results of the analyses.

The results of this study demonstrate that KXS and antidepressant drugs have promising therapeutic effects for the treatment of depression. KXS was more significant in improving depressive symptoms, the total effective situation, and side effects, and the results of the subgroup analysis also proved that *it has certain theoretical advantages in treating people in different living locations, emphasizing the individualized differences in TCM treatment, but this is also a limitation of this study, indicating that KXS is not necessarily applicable in different clinical settings or populations and that the therapeutic effects are not necessarily universally applicable. More caution is needed when promoting it to the clinic. The study on the general applicability of KXS should be strengthened in future studies, and more rigorously designed large-sample randomized controlled trials are also needed to gain a more comprehensive understanding of the efficacy and safety of KXS in different contexts, to strengthen the study on the mechanism of antidepressant effects of KXS, and to enhance the strength of the evidence.*

## 5. Conclusion

The results showed that KXS was comparable to or superior to antidepressants in the treatment of depression. The side effects of KXS are fewer than those of antidepressants. The data analysis showed that effectiveness and other indicators differed significantly by geographic area and dosage form, information that clinicians will find relevant in the practice of treating depression.

## Author contributions

**Conceptualization:** Jia-liang Li, Lin Lin, Min-min Wu, Long Wang.

**Data curation:** Jia-liang Li, Lin Lin, Jing-yu Zhang.

**Supervision:** Meng-ru Cao, Long Wang.

**Writing – original draft:** Jia-liang Li, Lin Lin, Yi-xin Zhang, Jing-yu Zhang.

**Writing – review & editing:** Min-min Wu, Meng-ru Cao, Long Wang.
